# Supplementary material for: The Impact of Corticosteroids on Mortality in Acute Exacerbations of Idiopathic Pulmonary Fibrosis: A Meta-Analysis
Source: Adv Respir Med. 2025 Mar 28;93(2):6. doi: 10.3390/arm93020006 (PMC12024352; doi:10.3390/arm93020006)
Supplement: Supplementary file 1 [file arm-93-00006-s001.zip › arm-3497329-supplementary.pdf]

Supplementary Table S1

Supplementary Table S1. Summary of the characteristics and results of the prospective randomized controlled trials (RCTs) included in this meta-analysis.

Study	Mortality Steroid (n)	Mortality Control (n)	Total Steroid (N)	Total Control (N)	Risk Ratio (RR) with 95% CI
Donahoe et al. (2015)	5	10	50	60	0.6 (0.75–0.99)
IPFCRN (2014)	30	35	100	110	0.94 (0.89–1.1)
Naccache et al. (2017)	60	65	120	130	1 (0.97–1.23)
Naccache et al. (2019)	50	55	110	120	0.99 (0.84–1.31)
